# Understanding time-growth rate relationship for slow-growing microbial cells in retentostat culturing systems

**DOI:** 10.1007/s00253-026-13817-x

**Published:** 2026-04-25

**Authors:** Beatriz Aranda, Juan M. Gonzalez

**Affiliations:** Instituto de Recursos Naturales y Agrobiologia de Sevilla, Consejo Superior de Investigaciones Cientificas (IRNAS-CSIC), Seville, Spain

**Keywords:** Continuous culture, Retentostat, Growth rate, Near-zero growth, Growth limitation, Nutrient-limiting growth

## Abstract

Generally, microorganisms in nature are severely growth-limited because of nutrient availability, competition, or physico-chemical parameters. To simulate these growing conditions, the use of continuous culturing systems has been proposed. One modification of these systems is the so-called retentostat. It corresponds to a system closed to cells and open to nutrients, and a nutrient is limiting growth. In this system, cells are forced to reduce progressively their growth rate as a result of increasing biomass sharing the same resources. In these systems, near-zero growth rates (corresponding to long doubling times) can be achieved within relatively reasonable time frames. Nevertheless, in these systems, the relationship between growth rate and time remains to be further analyzed. Herein, we evaluate a retentostat culturing system for different prokaryotic species. We established a general relationship between growth rate and incubation time. This allows us to easily estimate either the growth rate reached after a given incubation time or the time required to obtain cells at a specific slow growth rate. The results suggested progressive adaptation of cells at decreasing growth rates. This contribution simplifies estimates for incubation time-dependent growth rates and shows a retentostat as a species-independent culturing system where growth rates depend on time and dilution rate.

## Introduction

Standard growth of prokaryotes in a microbiological laboratory is generally performed in batch cultures. These cultures follow the typical series of growth phases for microorganisms: an initial lag phase, followed by an exponential phase at maximum growth, a stationary phase of growth due to exhaustion of the available nutrients, and a relatively long death phase showing a progressive decrease in cell number (Gonzalez and Aranda 2023; Navarro-Llorens et al. 2010; Roszak and Colwell 1987). Cells in each of those four growth phases present specific physiological characteristics according to the relationship of the cells with the available conditions (available nutrients and cultivation conditions) (Bergkessel et al. 2016; Ferenci 1999). In batch cultures, we can only estimate the growth rate of the cells at the midpoint of the exponential phase (Gonzalez and Aranda 2023). However, cells at very slow growth rates cannot be experimentally obtained in a batch system because of experimental and timing issues.

**Fig. 1:**
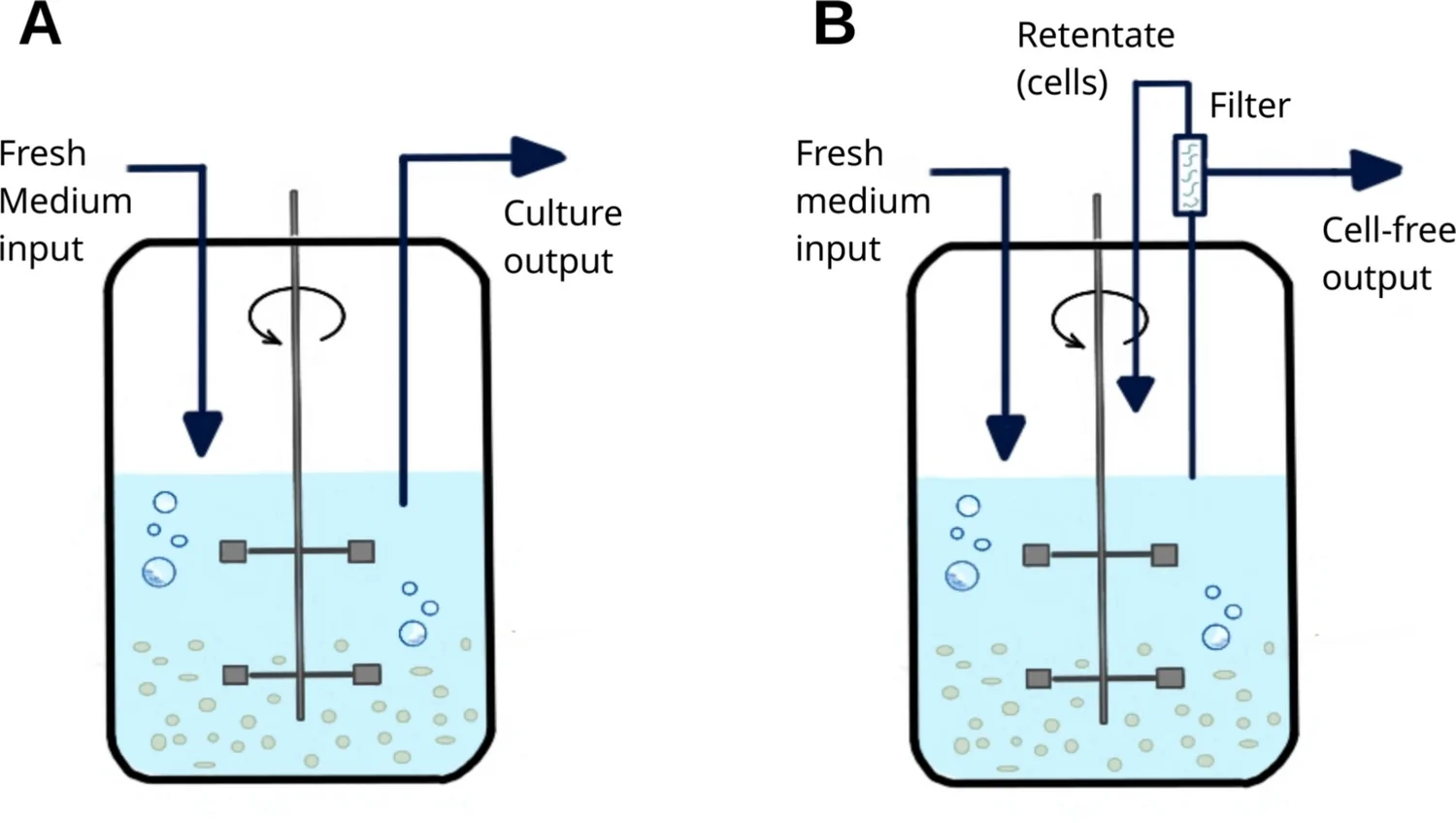
Schematic representations comparing the chemostat (**A**) and retentostat (**B**) culturing systems

In order to know the precise growth rate of the cells in a microbial culture, it is required to carry out continuous cultures. A chemostat is the typical culturing system used for this purpose. A chemostat is an open system for cells and nutrients (Fig. 1A). According to the reported growth model for chemostats (Greenman et al. 2022; Gresham and Hong 2015; Novick and Szilard 1950), the growth rate of the cultured cells is exclusively dependent on the established dilution rate. In a chemostat culturing system, cells are at a steady-state condition, and the growth rate of these cells is maintained constant as far as the conditions are preserved.

In nature, cells thrive under strictly limiting conditions (Demoling et al. 2007; Gonzalez and Aranda 2023; Morita 1997; Porter et al. 2025; Torsvik et al. 2002)due to a variety of factors, such as limited nutrient availability, competition, or other environmental and physico-chemical factors. Different authors have mentioned the huge diversity of microorganisms inhabiting our planet (Curtis et al. 2002; Dubey et al. 2006; Grundmann 2004; Tecon and Or 2017). Most of those microorganisms are difficult to culture using standard culturing methods (Epstein 2013; Joergensen and Wichern 2018; Martiny 2020). Different authors have reported that the majority of microorganisms thriving in nature live at very reduced growth rates (Gonzalez and Aranda 2023; Hoehler and Jørgensen 2013; Whitman et al. 1998). An extreme example of this scenario is the one of cells thriving in the deep subsurface presenting doubling times in the order of hundreds of years (Hoehler and Jørgensen 2013; Lever et al. 2015; Parkes et al. 1990; Whitman et al. 1998). These cells are believed to show very reduced, near-zero growth limited by the available nutrient supply. In the laboratory, in order to achieve cells growing at the so-called near-zero growth rates, a modification of a standard continuous culturing system is required (Boender et al. 2009; Ercan et al. 2015; Gonzalez and Aranda 2023; van Mastrigt et al. 2019; Overkamp et al. 2015; Tappe et al. 1996). This is a retentostat, which is a closed system for cells and open for nutrients (Fig.1B); cells are retained, progressively increasing in number at the same time that they must share the same nutrient resource (constant dilution rate) so cells are forced to grow progressively at slower rates along the incubation time (Gonzalez and Aranda 2023).

Cells at near-zero growth have been obtained for several prokaryotic species. Until now, the culturing system needs to be monitored along the incubation time by measuring cell biomass. At the end of the experiment, this allows us to estimate the growth rate that the cells had at different times during the incubation (Boender et al. 2009; Ercan et al. 2015; van Mastrigt et al. 2019; Overkamp et al. 2015; Tappe et al. 1996). Herein, we analyze the growth rates*versus* incubation time for several prokaryotic species (as examples) aiming to obtain generalized growth estimates in a retentostat culturing system. In this way, one will be able to determine a priori: the time required to obtain cells at a specific growth rate or the growth rate of cells after a pre-established incubation time period.

## Materials and methods

### Culture media and prokaryotic species

The growth of six prokaryotic species was evaluated in a retentostat culturing system. The strains and culture media used in this study are the following:

- *Acidobacterium capsulatum*, strain DSM 11244, was grown at 30 °C in medium DSMZ 269 containing (per liter): (NH_4_)_2_SO_4_ 2 g, KCl 0.1 g, K_2_HPO_4_ 0.5 g, MgSO_4_.7H2O 0.5 g, yeast extract 0.1 g, glucose 1 g. Final pH 3.5.
- *Azotobacter vinelandii*, strain DSM 366 was grown at 30 °C in medium DSMZ 3 composed of (per liter): glucose 5 g, mannitol 5 g, CaCl_2_.2H_2_O 0.1 g, MgSO_4_.7H_2_O 0.1 g, Na_2_MoO_4_.2H2O 5 mg, K_2_HPO_4_ 0.9 g, KH_2_PO_4_ 0.1 g, FeSO_4_.7H2O 10 mg, CaCO_3_ 5 g. Final pH 7.3.
  *Gluconacetobacter sacchari*, strain DSM 12717, was cultured at 30 °C in medium Sabouraud-2% Dextrose Broth containing (per liter): peptone 10 g, glucose 20 g, ethanol 2%. Final pH 6.8.
- *Methylobacterium organophilum*, strain DSM 18172, was cultivated at 30 °C in medium DSMZ 125 containing (per liter): KNO_3_ 1 g, MgSO_4_.7H_2_O 0.2 g, CaCl_2_.2H_2_O 0.02 g, Na_2_HPO_4_ 0.23 g, NaH_2_PO_4_ 0.07 g, FeSO_4_.7H_2_O 1 mg, CuSO_4_.5H_2_O 5 mg, H_3_BO_3_ 10 μg, MnSO_4_.5H_2_O 10 μg, ZnSO_4_.7H_2_O 70 μg, MoO_3_ 10 μg, methanol 5 ml. Final pH 6.8.
- *Sulfolobus acidocaldarius*, strain DSM 639, was grown at 75 °C and pH 3.0 in medium DSMZ 88 containing (per liter): (NH_4_)_2_SO_4_ 1.3 g, KH_2_PO_4_ 0.28 g, MgSO_4_.7H_2_O 0.25 g, CaCl_2_.2H2O 0.07 g, FeCl3.6H2O 0.02 g, yeast extract 1 g, Allen’s trace element solution 10 ml. Allen’s trace element solution contains (per liter): MnCl_2_.4H_2_O 180 mg, Na_2_B_4_O_7_.10H_2_O 450 mg, ZnSO4.7H2O 22 mg, CuCl_2_.2H_2_O 5 mg, Na_2_MoO_4_.2H_2_O 3 mg, VOSO_4_.2H_2_O 3 mg, CoSO_4_.7H_2_O 1 mg with final pH 2.
- *Thermus thermophilus*, strain HB8, DSM 579, was grown at 70 °C in medium DSMZ 878 which contains (per liter): yeast extract 1 g, tryptone 1 g, nitrilotriacetic acid 100 mg, CaSO_4_.2H_2_O 40 mg, MgCl_2_.6H_2_O 200 mg, 0.01 M Fe citrate 0.5 ml, trace element solution 0.5 ml, phosphate buffer 100.0 ml. Final pH 7.0. The trace element solution contains (per liter): H2SO4 (12 N) 0.5 ml, MnSO_4_.H_2_O 2.28 g, ZnSO_4_.7H_2_O 0.5 g, H_3_BO_3_ 0.5 g, CuSO_4_.5H_2_O 25 mg, Na_2_MoO_4_.2H_2_O 25 mg, CoCl_2_.6H_2_O 45 mg. The phosphate buffer contained (per liter): KH2PO4 5.44 g, and Na_2_HPO_4_.12H_2_O 43 g.

### Retentostat culturing system

A vessel containing 1.8 l culture was fed with fresh culture medium at a flow rate of 45 ml h^−1^. This resulted in a growth rate of 0.025 h^−1^ in the chemostat culture. This continuous culture represented the starting conditions (time 0 days) for the retentostat culturing system which maintained the same flow rate (Fig. 1). The retentostat system was established as previously reported (Boender et al. 2009; Overkamp et al. 2015)using a tangential flow filtration set up with a 0.2 μm-pore-diameter hollow fiber filter (SpectrumLabs, Irving, TX, USA) to return the cells to the vessel. The pH was monitored by a pH electrode and automatically adjusted during the incubation time pumping in HCl or NaOH (1 M) as needed. Samples were collected aseptically along the incubation time to determine total cell number and total biovolume. Total cell number was enumerated by using a Neubauer chamber and cell biovolume, as an approach to cell biomass, was calculated from the cell dimensions (area and perimeter) following (Massana 1997). Growth rate estimates over the incubation time were calculated following the indications by different authors (Boender et al. 2009; Overkamp et al. 2015) with some modifications as described in the Annex.

### Statistics

Regressions were performed using the spreadsheet built data analysis (LibreOffice Calc). Linear and non-linear regression analyses were used to establish the relationships between prokaryote biovolume accumulated and incubation time and between growth rate and the inverse of time according to the equations shown below (see Annex). Significant difference from the 1:1 slope was estimated from least-square Model II regression analysis as described by (Sokal and Rohlf 1995).

## Results and discussion

**Fig. 2:**
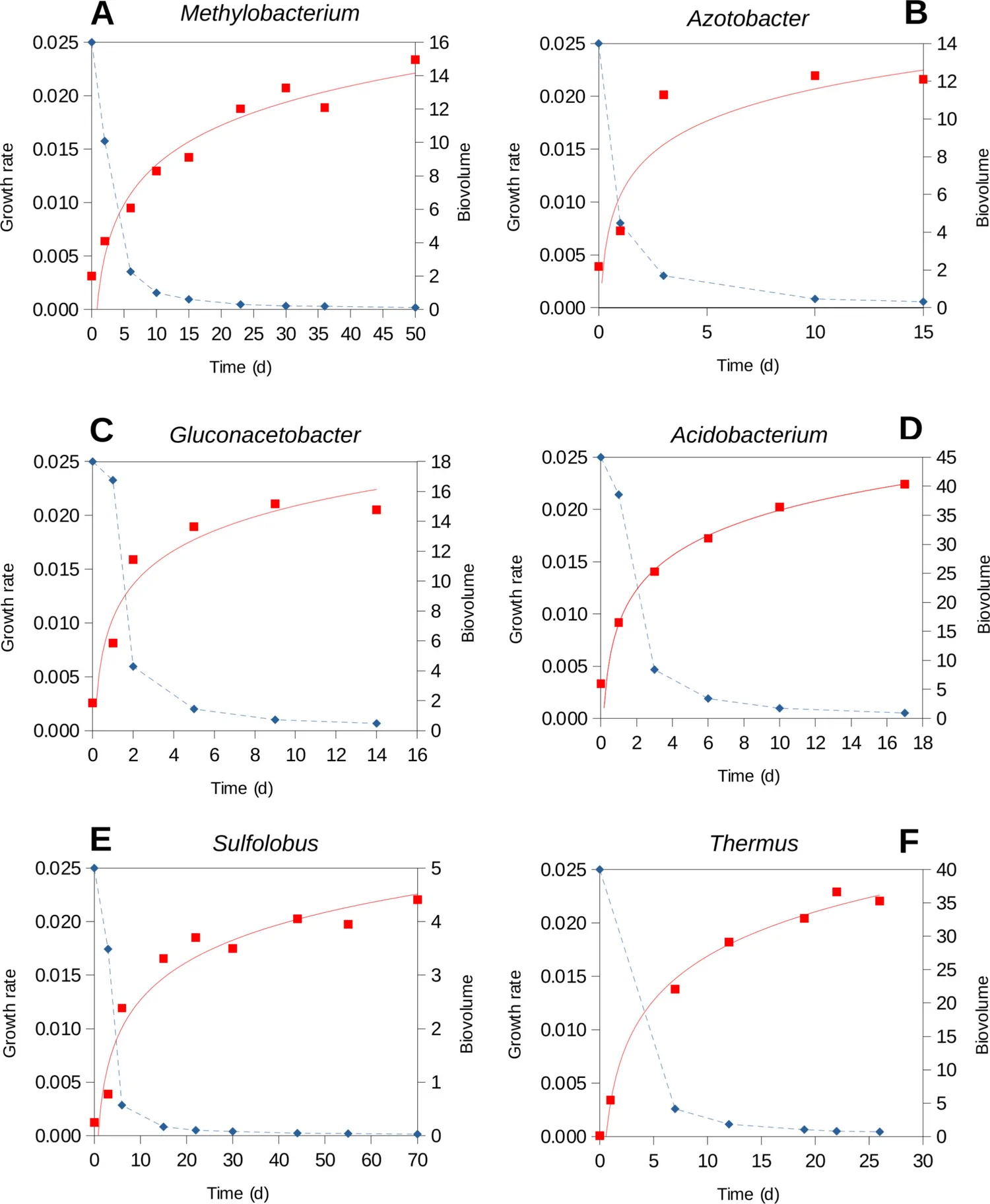
Plots of the biovolume accumulated (red squares) and growth rate estimates (blue diamonds linked by a dashed line) over time during the incubation in a retentostat culturing system for different prokaryotic species. Growth rates are estimated by sampling the retentostat culture over time and fitting the data points to the curve model (red continuous line) shown in Eq. 5 (Annex). **A** *Methylobacterium organophilum*. **B** *Azotobacter vinelandii*. **C** *Gluconacetobacter sacchari*. **D** *Acidobacterium capsulatum*. **E** *Sulfolobus acidocaldarius*. **F** *Thermus thermophilus*

**Fig. 3:**
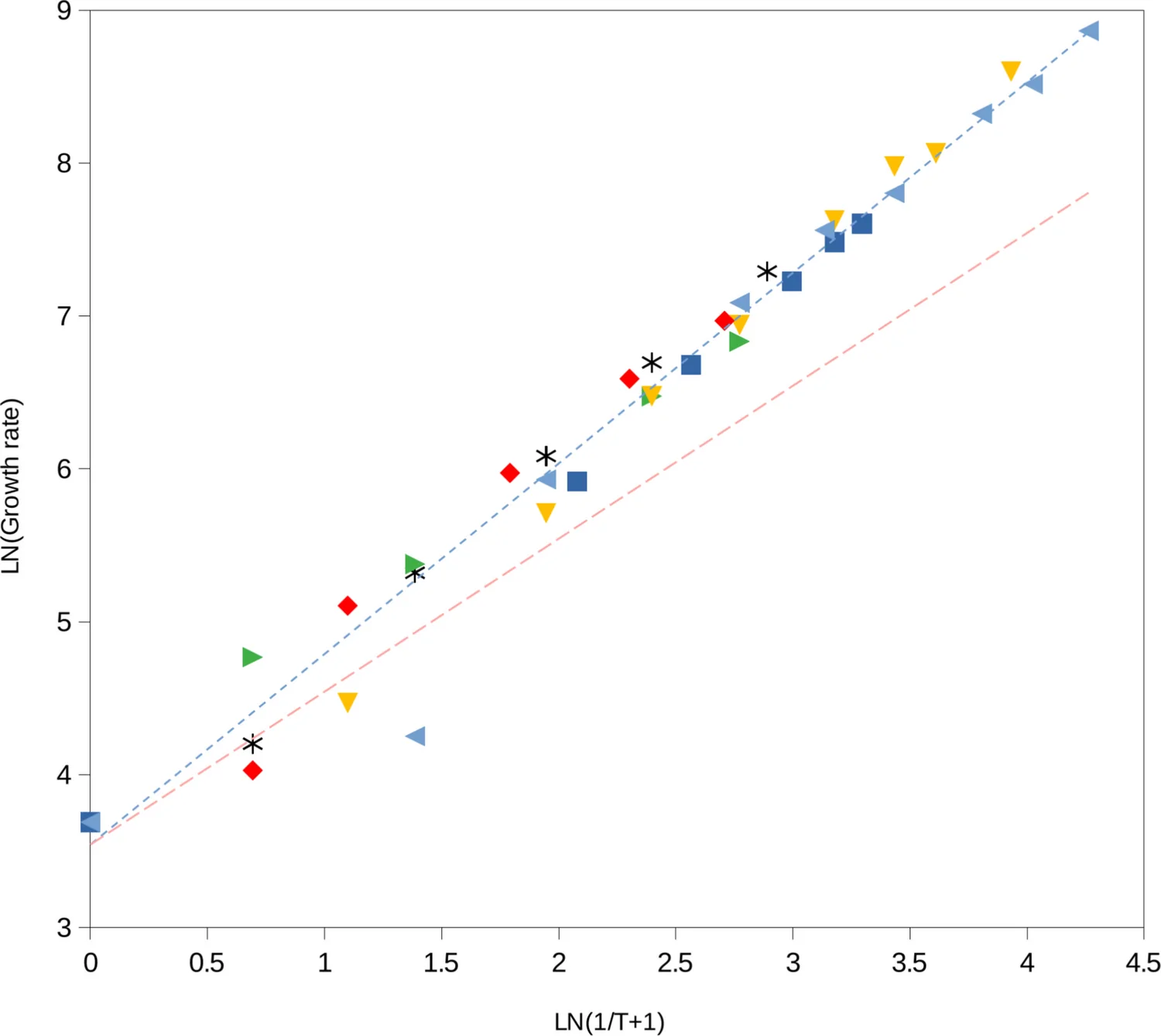
Relationship between growth rate and incubation time for a retentostat culturing system at a dilution rate 0.025 h^−1^ showing the results from different prokaryotic species: *Acidobacterium capsulatum* (Black asterisks), *Azotobacter vinelandii* (light blue triangles pointing left), *Gluconacetobacter sacchari* (red diamonds), *Methylobacterium organophilum* (orange triangles pointing down), *Sulfolobus acidocaldarius* (green triangles pointing right), *Thermus thermophilus* (dark blue squares). Growth rates are in h^−1^ and time in days. Regression line (blue dashed line) corresponds to the best fit reported in this study: Ln(μ) = 3.54264 + 1.24636 × Ln(1/(t + 1)) (*r*^2^ = 0.98, *n* = 41, *P* < 0.001) (Eq. 1). The line corresponding to a slope *n* = 1 is shown as reference (red dashed line)

In this study, different prokaryotic species have been cultured in a retentostat culturing system starting from cells at a growth rate of 0.025 h^−1^. The increase of biomass and the decrease of growth rate over the incubation time in retentostat mode for each prokaryote tested in this study are shown in Fig. 2. Those growth rate data points for different prokaryotes are shown in Fig. 3. Data fitting to the predicted equation (see Annex) resulted in Eq. 1:

1 $$\begin{array}{cc}\mathrm{Ln}\;\mu=3.54264+1.24636\;\mathrm{Ln}\;\left(1/\left(\mathrm t+1\right)\right)&\left(\mathrm r^2=0.98,\;\mathrm n=41.\;\ P<0.001\right)\end{array}$$

where μ represents the growth rate (h^−1^), t the incubation time (d), and n the regression coefficient (slope) of the line. The estimates of growth rates and doubling times for cells grown in that retentostat are summarized in Table 1. These results show that long incubation times can lead to obtaining cells with high doubling times, reaching values in the order of months or years.

**Table 1:** Expected values of growth rates and their corresponding doubling times over the incubation

Time (d)	Growth rate (h^−1^)	Doubling time (h)	Doubling time (units) *
0	0.025000	27.7	1.1 d
2	0.007358	94.2	3.9 d
5	0.003101	223.5	9.3 d
10	0.001457	475.7	19.8 d
15	0.000913	758.8	31.6 d (1.0 mo)
20	0.000651	1065.0	44.4 d (1.5 mo)
30	0.000401	1730.4	72.1 d (2.4 mo)
50	0.000215	3218.3	134.1 d (4.5 mo)
100	0.000092	7541.9	314.2 d (10.5 mo)
150	0.000056	12,449.9	518.7 d (17.3 mo; 1.4 y)
200	0.000039	17,782.3	740.9 d (24.7 mo; 2.1 y)
300	0.000024	29,414.7	1225.6 d (40.9 mo; 3.4 y)
365	0.000018	37,531.7	1563.8 d (52.1 mo; 4.3 y)

*h hours, *d* days, *mo* months, *y* years

The slope of Eq. 1 would be expected (see Annex) to be *n* = 1. The results (Fig. 3; Eq. 1) showed a best fit for *n* = 1.246 which was significantly different from the expected slope *n* = 1 (*P*< 0.05). As a result, cells appeared to be growing in the retentostat a bit faster than theoretically predicted. Different potential explanations could justify this result. Experimental error during culturing and quantification, for instance, a decrease in cell viability or a poor filtration of the retentate during the growth in retentostat mode, would have likely implied a reduction in growth. None of these two possibilities were observed, and the cultures maintained cell culturability and no cells were detected in the discharged cell-free medium. A most likely factor for an increase in the slope (n) could be attributed to a progressive adaptation of the cells to severe nutrient-limiting conditions. Stress responses in prokaryotes and adaptations through exposure to limiting and adverse conditions have been frequently reported (Moreno-Gámez 2022; Morita 1997; Plum 2026; Santana et al. 2026; Ultee et al. 2019). Nevertheless, to our knowledge, no clear relationship to growth rate estimates has been experimentally proposed in retentostat cultures. This result indicates that cells beyond day 5 in the retentostat culturing system (equivalent to a growth rate of 0.003 h^−1^; Table 1) appeared to have experienced an improvement in their adaptation to severe nutrient-limiting growth. Before this time point (day 5) during the incubation some variability among the species is observed suggesting species-specific adaptive mechanisms or time responses. Progressive adaptive mechanisms to severe nutrient-limiting conditions have been reported (Gray 2019; Phaiboun et al. 2015)although they need further evaluation, for instance by whole-cell gene expression transcriptomic analysis (Gray et al. 2015; Overkamp et al. 2015; Santana et al. 2026). This opens new perspectives to understand oligotrophy and the adaptive mechanisms involved in prokaryotes facing nutrient scarcity.

In the present study, we have predicted (see Annex) and established experimentally (experimental results) a growth model for a retentostat culturing system. We have shown that the growth rate is linearly related to the inverse of incubation time, and vice versa. The culturing conditions for a determined retentostat set up are based on the dilution rate, similarly to continuous culturing systems using chemostats (Gonzalez and Aranda 2023; Harmand et al. 2017; Smith and Waltman 2009). As a consequence, the incubation time required to obtain a desired growth rate, or the growth rate to be reached after a specified incubation time, can be a priori estimated. This will greatly simplify the use of these culturing systems avoiding the need for time-consuming measurements of biovolume or biomass accumulation, total cell counts, nutrient utilization, and growth along the incubation of the cells in the culture.

In nature, the growth of microorganisms is generally limited by nutrient availability; physico-chemical conditions (e.g., temperature, pH, and humidity); and biotic components of the microbiome such as competition for limited resources (Demoling et al. 2007; Morita 1997; Torsvik et al. 2002). Nevertheless, most microbiological studies have been carried out using laboratory cultures at optimum (maximum) growth rates (Gonzalez and Aranda 2023). The use of chemostats and retentostats has allowed a step further to analyze the physiology and, in general, microbial cell responses under specific growth rates, including slow growth and near-zero growth rates (Boender et al. 2009; Ercan et al. 2015; Gonzalez and Aranda 2023; van Mastrigt et al. 2019; Overkamp et al. 2015; Tappe et al. 1996). In fact, numerous microorganisms have been reported to be able to grow only at very slow growth rates. These slow growers are often masked by the growth of the fast-growing cells when natural samples are cultured in traditional laboratory rich medium cultures (Torsvik et al. 2002). In an attempt to select for slow microbial growers present in natural samples and to obtain microbial cells at a specified growth rate in the laboratory, the capability of calculating growth rates over the incubation time in a retentostat culturing system is of high interest to achieve those goals. These results will contribute to increase the proportion of identified cultured bacteria from nature (Epstein 2013; Martiny 2020)among the vast prokaryotic diversity in our planet (Curtis et al. 2002; Joergensen and Wichern 2018; Whitman et al. 1998).

Another use of a retentostat culturing system is, for example, the analysis in depth of the behavior of prokaryotic cells at near-zero growth (Boender et al. 2009; Ercan et al. 2015; Gonzalez and Aranda 2023; Overkamp et al. 2015)and so one can study these cells’ behavior, whole-cell gene expression and progressive adaptive mechanisms after long-term exposure to severe nutrient scarcity. In biotechnology, growth optimization can lead to increased process efficiency; maximum productivity can be obtained by decreasing the substrate to be derived for growth (Coltman et al. 2024; Ercan et al. 2015). For instance, this can be achieved by using cells at near-zero growth rates in a retentostat culturing system.

Previous studies (Gonzalez and Aranda 2023; Harmand et al. 2017; Smith and Waltman 2009) had clearly established that in a chemostat culturing system (i.e., in a continuous culture) the growth rate of cells is only dependent on dilution rate. Estimates of growth in a retentostat are more complex, and cell biomass had to be quantified over the incubation time to fit data and calculate growth rates. From our results, the capability to easily estimate the growth rate of cells after a specified incubation time in a retentostat culturing system greatly facilitates the analysis of cell behavior as a function of growth. Similarly, the growth model in a retentostat system significantly assists in estimating the time required to obtain cells at specific growth rates. However, attention should be given to adjusting growth parameters (such as dilution rate and the overall setup of the retentostat culturing system) to calculate or “calibrate” the retentostat system incubation time-growth rate relationship. Once this step is achieved, the use of a retentostat culturing system is greatly facilitated because measurements of cell biomass and cell counts are no longer needed to estimate growth. After those preliminaries have been established, cell growth rate can be estimated from incubation time in the retentostat culturing system.

It is important to remember that cells at near-zero growth, as achieved in retentostat culturing systems, are growing cells (Coltman et al. 2024; Ercan et al. 2015; Gonzalez and Aranda 2023; Overkamp et al. 2015). This is a completely different scenario than the case of cells under survival by adverse conditions or at late-stationary phase of growth (Gonzalez and Aranda 2023). In these last cases, cells can be in non-growing states; they might be at declining phases of their living cycle or attempting to adapt to the newly found conditions, showing a response to adversity which is inhibiting them from growing or even persisting. A great variability of the individual cell status in a population might occur under these adverse conditions, and their specific growth rates remain unknown (Gonzalez and Aranda 2023). In retentostat culturing systems, prokaryotic cells encounter growth limitation, but they are able to grow although at low rates; over time, their growth rate will progressively decrease, leading to near-zero growth rates. From our results, the required incubation time can be assessed before the experimental work is to be performed (Fig.3 and Table 1).

Near-zero growth rates represent a general situation for most prokaryotic species. The most typical case is believed to occur in the deep subsurface, but prokaryotes are growth-limited in most natural environments. The growth of cells is strictly limited by resources and competition in their environments (Hoehler and Jørgensen 2013; Morita 1997; Parkes et al. 1990; Whitman et al. 1998). The generalized use and understanding of the retentostat culturing systems will significantly contribute to studying the physiology of slow-growing prokaryotes from a variety of environments and biotechnological processes. These culturing systems and their quantitative analyses will facilitate deciphering cell behavior and will contribute to better understanding the phenotype and adaptive strategies of prokaryotic cells growing at very low rates.

## Data Availability

Data generated in this study is available at the repository https://digital.csic.es/ linked to this study.

## Annex

Estimates of growth rates of prokaryotic cells are not easy to obtain. Specific conditions need to be followed so that growth rates can be calculated. Below, a summary is presented on how growth rates can be calculated in batch cultures, chemostat, and retentostat culturing systems.

### A batch culture

In a batch culture, growth can only be reliably estimated during the exponential phase of growth and more precisely at the mid point of the exponential phase of growth which is represented by the inflection point of the growth curve (Gonzalez and Aranda, 2023. Different sigmoidal models have been proposed such as the Logistic and Gompertz models among some others (Zwietering et al. 1990. A simplified way to estimate the growth rate during an exponential growth is by plotting Ln (cell number ml^−1^) *vs*. incubation time. During the linear portion of the curve (the exponential phase of growth in a batch culture), the slope represents the growth rate of the culture and this can be approached by a regression line according to the equation (Eq. 2):

2 $$\mathrm{Ln}\left({\mathrm N}_{\mathrm t}\right)=\mathrm{Ln}\left({\mathrm N}_{\mathrm o}\right)+\;\mathrm\mu\;.\;\mathrm t$$

where N_t_ and N_0_ are the number of cells at time 0 and t, μ is the growth rate, and t is incubation time. The growth rate (μ) corresponds to the slope or regression coefficient of that line.

Growth rate and doubling time can be calculated from the following equations (Eq. 3):

3 $$\begin{array}{ccc}\mathrm\mu=\mathrm{Ln}\left(2\right)\;/\;{\mathrm T}_{\mathrm d}&\mathrm{and}&{\mathrm T}_{\mathrm d}=\mathrm{Ln}\left(2\right)\;/\;\mathrm\mu\end{array}$$

where μ is the growth rate (as above) (generally as h^−1^) and T_d_ is the doubling time or generation time (generally as hours or days).

### Continuous culture – chemostat

The estimate of growth rates in continuous culturing systems have been reported in numerous publications. Following, a brief description of growth rate estimation strategies for continuous cultures in chemostat mode is presented as previously described (Harmand et al. 2017, and the proposed approach to estimate growth rate during the incubation time is deduced for a retentostat culturing system.

In a continuous culture (i.e., chemostat), the growth rate is given by the flow of fresh nutrients into the culture vessel, which equals the flow out of culture. This represents the dilution rate in a continuous culture which equals the growth rate of the microorganisms in the chemostat. In this situation, cell growth equals resource supply because growth is being limited by nutrient availability in the culturing system. Under these conditions, cells remain at a steady-state growing at a constant rate. In this set up, cell growth is not influenced by other factors. In a chemostat, the dilution rate is the ratio of the flow of fresh medium by total volume of the culture (Gonzalez and Aranda, 2023.

### Retentostat

The estimation of growth rate along the incubation time in a retentostat culturing system has been previously described (Boender et al. 2009; Overkamp et al. 2015. The kinetic of growth in a retentostat culture can be estimated from the biomass balance of the following equation (Eq. 4):

4 $${\mathrm{dC}}_{\mathrm x}/\mathrm{dt}=\;\mathrm\mu\;{\mathrm C}_{\mathrm x}$$

where C_x_ represents biomass concentration (which can be expressed as g l^−1^); t is the incubation time of the retentostat culture; dC_x_/dt is the change of biomass concentration over incubation or, in other words, the derivative of cell biomass over time; and μ represents the growth rate (h^−1^) which is the variable we want to estimate.

From Eq. 4, we can express the change of biomass concentration over time by Eq. 5. In Eq. 5 variation of cell biomass over time follows a logarithmic model where growth is increasingly limited along the incubation time in a retentostat culturing system as far as nutrient supply and incubation conditions are maintained constant during the incubation in continuous cultivation systems. This logarithmic model fits the progressive limitation of cellular growth in the retentostat (Fig. 2). This logarithmic model represents a correction to previous publications (Boender et al. 2009; Overkamp et al. 2015 where an exponential model was erroneously mentioned to fit biomass increase data over incubation time in a retentostat. After this correction is applied, cell biomass as a function of time can be expressed by the following equation (Eq. 5):

5 $${\mathrm C}_{\mathrm x}=\mathrm a\;\mathrm{Ln}\;\left(\mathrm t+1\right)\;+\;\mathrm b$$

where C_x_ and t are, as above, biomass and time, respectively. The parameters a and b are estimated from the curve resulting by plotting C_x_ vs. t in the retentostat culture. They are calculated from non-lineal regression of experimental data resulting from over time measurements of cell biomass in a retentostat culturing system. By using *t* + 1, Eq. 5 has solution at *t* = 0 indicating that C_x_ equals b. The value of b represents the biomass existing in the culture under chemostat conditions at the dilution rate setup in the system and at *t* = 0 equals C_x_ in the retentostat. A chemostat culture is a required step previous to initiate the retentostat culturing mode in the system.

Equation 5 can be linearized plotting C_x_ *vs*. Log(*t*+1) by using a linear regression analysis. Today, most spreadsheets (i.e., Microsoft Excel or LibreOffice Calc) can perform both logarithmic and lineal fitting of data points so both the Equation and plotting strategies are easy to use.

From Eq. 4, one can express growth rate (μ) as (Eq. 6):

6 $$\mathrm\mu=\left({\mathrm{dC}}_{\mathrm x}/\mathrm{dt}\right)\;/\;{\mathrm C}_{\mathrm x}$$

which means that the growth rate can be calculated from the estimation of the derivative of the curve of cell biomass over the incubation time. This derivative (Eq. 7) is given by

7 $$\left({\mathrm{dC}}_{\mathrm x}/\mathrm{dt}\right)={\mathrm C}_{\mathrm x}'=\mathrm a\left(1/\left(\mathrm t+1\right)\right)$$

where C_x_ represents (as above) a measurement of cell biomass; t is the incubation time (d); C_x_′ is the derivative to the curve in t or the slope to the curve at each time point (t); and, a is the regression coefficient obtained by fitting cell biomass *vs*. time data, as above (Eq. 5).

For a specific set up of a retentostat culturing system and a given dilution rate, as shown in this study (Fig. 3), the change of growth rate as a function of time follows the same variation for any microorganism to be tested. This change of growth rate remains constant over time which allows to calculate the growth rate at any given t along the incubation in a retentostat culturing system. As a consequence, we are able to estimate the growth rate (*μ*) of cells as a function of incubation time (*t*) in this retentostat system. This estimate can be obtained by lineal regression fitting the experimental data of growth rate (from Eq. 6) plotted against the inverse of incubation time (1/days) following Eqs. 6 and 7 (Eq. 8):

8 $$\mathrm\mu=\left({\mathrm{dC}}_{\mathrm x}/\mathrm{dt}\right)\;/\;{\mathrm C}_{\mathrm x}=\left(\mathrm a/{\mathrm C}_{\mathrm x}\right)(1/\left(\mathrm t+1\right))$$

which can be expressed in logarithm form with the straight line (Eq. 9)

9 $$\mathrm{Ln}\;\mathrm\mu=\mathrm{Ln}\;\left(\mathrm a/{\mathrm C}_{\mathrm x}\right)\;+\;\mathrm{Ln}\;\left(1/\left(\mathrm t+1\right)\right)$$

and this expression can be experimentally estimated by linear fitting according to the equation (Eq. 10)

10 $$\begin{array}{ccc}\mathrm{Ln}\;\mathrm\mu=\mathrm A+\mathrm n\;\mathrm{Ln}\;\left(1/\left(\mathrm t+1\right)\right)&\mathrm{or}&\mathrm{Ln}\;\mathrm\mu=\mathrm A-\mathrm n\;\mathrm{Ln}\;\left(\mathrm t+1\right)\end{array}$$

where n represents the regression coefficient (slope) of the straight line resulting from fitting experimental data as a natural logarithm of growth rates *versus* natural logarithm of 1/(*t* + 1). The slope n would be expected to be 1 if no additional factors would be influencing bacterial growth under nutrient-limiting conditions in a retentostat culturing system. Nevertheless, experimental error and biological variables could result in deviations from this value (see Results and Discussion).

The linear fit resulting from Eq. 10 (Eq. 1) is specific for a singular retentostat culturing system with defined conditions (dilution rate and filtration system set up).

Having a previously tested retentostat culturing system under specified allows to determine how long the cells should be incubated in the retentostat to achieve cells at a specified growth rate. Similarly, one can determine the growth rate of cells after a specific incubation time period and retrieve those cells for experimental evaluations. This relationship is universal for a given retentostat setup independently of prokaryotic species and culture medium composition.
